# Hæmopneumothorax

**Published:** 1900-02-24

**Authors:** 


### HÆMOPNEUMOTHORAX.

At the last meeting of the Clinical Society several cases were reported in which that comparatively rare condition, hæmopneumothorax, existed. The first was one described by Dr. Rolleston in which a man, aged 21, whose past history showed nothing but some dyspepsia, after a couple of days of diarrhœa and abdominal pain was suddenly seized with intense pain in the right hypochondrium, radiating to the umbilicus and right shoulder. When admitted to St. George's Hospital 25 hours later he appeared moribund, but after a time rallied, and next day was found to have signs of pneumothorax on the right side, with considerable displacement of the heart. Paracentesis of the right side of the chest brought out air and blood at considerable pressure. He died eight days after the onset of the acute pain. At the necropsy the right pleura contained 60 oz. of blood and no air. No cause for the blood or the gas in the pleura was found.

Dr. Newton Pitt reported a case in which a young man, aged 18, four days after a slight illness with sore throat which confined him to the house for one day, was attacked with pain in the right side while walking out. When seen he had symptoms of pneumothorax, but on inserting a Southey's tube in the sixth space in the axilla, to relieve pressure, some ounces of blood escaped. After that the tube became blocked with clot, and on being reinserted further forward blood and air bubbled out. The patient was not relieved by the operation, and died the same night. At the necropsy the right pleura was found to contain eight pints of fluid blood, and as much clot as filled the hands three times, besides a considerable quantity of air. No perforation or injury could be discovered, nor was there a trace of tuberculous, inflammatory, or necrotic lesion in the lung; but there was an emphysematous bulla about half-an-inch in size torn open near the apex of the lung, and attached to this was a ruptured adhesion, the size of a knitting-needle, from which the blood might have come, but no aneurysmal pouch or large patent vessel was to be found. Dr. Kingston Fox mentioned a case in which a healthy and robust man, aged 44, was seized with symptoms of pleurisy with effusion, which on the tenth day was tapped, about 15 ounces of dark blood being taken away. Three days afterwards the fluid had increased, and there was pneumothorax, and 44 ounces of blood with much air was taken away. This patient quite recovered after a voyage to the Cape and back. He had previously been working in insanitary surroundings, and three other clerks in the office broke down with lung trouble within six months, so his case was probably of septic origin.

Several other instances of hæmo- or pneumohæmothorax were mentioned in the discussion, and Sir Dyce Duckworth said that he thought the sequence of events in such cases usually was rupture of the lung, pneumothorax, hæmorrhage, all being due to tuberculosis. It seems clear, however, from the careful examination made in the cases described by Dr. Rolleston and Dr. Newton Pitt, that this cannot always be the case, unless we are to accept as possibly tuberculous cases in which no signs of that condition can be discovered either before or after death. It is curious how little notice is taken of hæmothorax in the text-books. Evidently when it occurs as a main condition, that is not as a mere bloodiness of some other form of effusion, blood in the pleura must be regarded as a very serious accident demanding a most cautious prognosis. The treatment also appears to be a matter of much anxiety. It is difficult to avoid the conclusion that tapping the pleura and the consequent alterations of the relations of the part must in some such cases seriously add to the probability of a hæmorrhage, which may perchance have come to an end, being set up afresh. On the other hand it can hardly be hoped that the mere pressure of blood in the pleura will stop further effusion from taking place. Such a thing is contrary to ordinary surgical experience in regard to hæmorrhage in other parts. Again, the condition of the patient, owing to the pneumothorax, may be such as urgently to demand relief. At any rate it would seem clear that if the pleura is to be opened this should be done so as to relieve tension, but not by aspiration.

